# Coronary Artery Bypass Grafting in a Patient with Dextrocardia with Situs Inversus

**DOI:** 10.1155/2020/8885881

**Published:** 2020-12-14

**Authors:** Kaitlin E. Woods, J. W. Awori Hayanga, Daniel Sloyer, Roy E. Henrickson, Lawrence M. Wei, Heather K. Hayanga

**Affiliations:** ^1^Department of Medical Education, West Virginia University, Morgantown, WV, USA; ^2^Department of Cardiovascular and Thoracic Surgery, West Virginia University, Morgantown, WV, USA; ^3^Division of Cardiovascular and Thoracic Anesthesiology, Department of Anesthesiology, West Virginia University, Morgantown, WV, USA

## Abstract

Dextrocardia involves embryologic malformations leading to a right hemithorax heart with rightward apex. Situs inversus encompasses all viscera in mirrored position. A 76-year-old male with dextrocardia with situs inversus presented for coronary artery bypass grafting due to a non-ST elevation myocardial infarction. Management was altered accordingly. Electrocardiography leads and defibrillator pads were reversed. A left internal jugular vein central venous catheter provided direct access to the right atrium. Transesophageal echocardiography confirmation of aortic and venous cannulation required turning the probe right for the right-sided aorta and left for liver visualization, respectively. Proactive surgical and anesthetic management was imperative for the successful and uneventful outcome for this patient.

## 1. Introduction

Dextrocardia encompasses intrinsic cardiac embryological malformations involving a right hemithorax heart with a caudally and rightward directed apex. This differs from cardiac dextroposition in which the heart is rightward secondary to lung or other mediastinal structural abnormalities. Dextrocardia is often associated with other congenital and acquired defects that may have clinical implications [1]. Situs inversus describes a condition characterized by transposition of all thoracic and abdominal viscera [2]. According to an article published in the *Journal of Anesthesia*, dextrocardia was previously thought to be very rare and often clinically insignificant [1]; however, with the advancement of medical imaging, cardiac surgery, and critical care, an increased number of patients with congenital heart defects are surviving into adulthood, and this condition is being detected more frequently [3]. In this population, potentially associated congenital anomalies and acquired comorbidities must be considered to determining necessary alterations in surgical and anesthetic technique and management to take into account anatomic variation. We describe a patient with dextrocardia with situs inversus who underwent coronary artery bypass grafting and the considerations and variations in anesthetic management provided for a successful surgery and perioperative course.

### 1.1. Case Presentation

**Figure 1:**
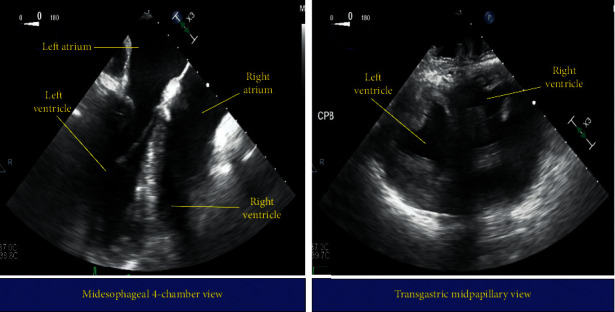
Midesophageal 4-chamber (left) and transgastric midpapillary transesophageal (right) echocardiography images.

A 76-year-old male with a past medical history of hypertension, hypercholesterolemia, hypothyroidism, chronic obstructive pulmonary disease, diabetes mellitus type two, obstructive sleep apnea, benign prostatic hyperplasia, cerebral vascular accident with residual right-sided weakness, and dextrocardia with situs inversus presented with an abnormal nuclear stress test. Additionally, he had a family history notable for colon cancer and myocardial infarction in his mother and a thrombotic event in his father. He had no history of significant alcohol use, tobacco use, or illicit drug use, and his past surgical interventions were not cardiovascular in nature. He originally presented at an outside hospital following a syncopal event with a 45-second loss of consciousness postmicturition. At the time, he was thought to have orthostatic hypotension, and his tamsulosin was discontinued. He also underwent a nuclear stress test for the syncopal event due to additional complaints of worsening dyspnea on exertion. Nuclear stress testing demonstrated a moderate-sized reversible anterior wall myocardial perfusion defect and a moderate-sized reversible inferolateral wall myocardial perfusion defect. He was transferred to our hospital for further evaluation of symptoms and possible left heart catheterization. Cardiac catheterization revealed the already diagnosed dextrocardia along with a new diagnosis of severe ostial left anterior descending (LAD), mid-LAD, and ostial diagonal coronary artery disease. Transthoracic echocardiography (TTE) showed trace mitral valve regurgitation, mild aortic valve regurgitation, and an ejection fraction of 60%. His underlying dextrocardia with situs inversus caused diagnostic challenge with these tests as his angiographic, radiologic, and echocardiographic images were mirror images of normal cardiac anatomy. Aggressive risk factor modification was initiated, and a consult was placed to cardiothoracic surgery for evaluation for potential coronary artery bypass grafting (CABG). He underwent three-vessel CABG on day 7 of his hospitalization with alterations in management to account for his known dextrocardia with situs inversus. The electrocardiography leads and defibrillator pads were placed in reverse orientation. A left internal jugular vein central venous catheter was placed to provide direct access to the right atrium rather than standard right-sided central venous access. The arterial line was placed in the right radial artery as opposed to the institutional standard left radial artery to accommodate for the position of the surgeon during the operation. Transesophageal echocardiography (TEE) evaluation again confirmed dextrocardia (Figure 1). To obtain standardly recognized TEE images, the omniplane was adjusted to 180° for the midesophageal four-chamber view. By subtracting 60°, 90°, and 120°, midesophageal midcommissural, two-chamber, and long-axis views were then obtained. Additionally, TEE confirmation of aortic and venous cannulation required turning the probe right for the right-sided aorta and left for liver visualization, respectively. Aside from these alterations in approach, induction, intubation, and anesthetic management were conducted in normal fashion and were uneventful. He remained intubated and was taken to a cardiac intensive care unit postoperatively. A thorough sign-out was given to the intensive care unit staff to ensure understanding of his anatomy and intraoperative course. He was weaned from the ventilator and extubated on postoperative day 1 and transferred to the step-down unit on postoperative day 2. His postoperative course was complicated by urinary retention and an elevation in creatinine that trended down prior to discharge. He was discharged home in stable condition with a urinary catheter in place on postoperative day 6 that was to be removed as an outpatient. Both the patient and the responsible physicians were satisfied with the successful outcome.

## 2. Discussion

Dextrocardia specifically describes the positioning of the heart within the thoracic cavity [4]. Dextrocardia encompasses intrinsic cardiac embryological malformations involving a right hemithorax heart with a caudally and rightward directed apex, differing from dextroposition, which is a resultant rightward heart caused by other mediastinal conditions or anatomic abnormalities [1].

**Figure 2:**
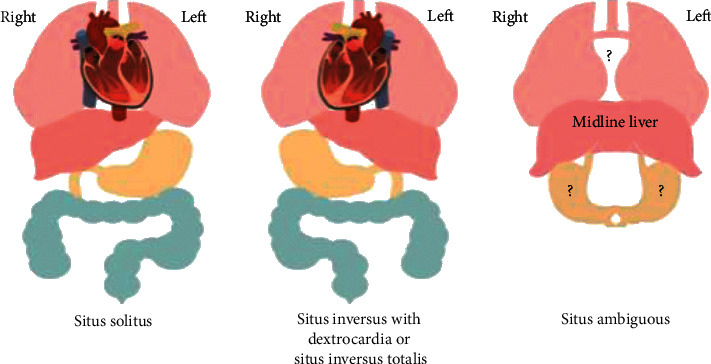
Illustration of situs solitus, situs inversus totalis, and situs ambiguous.

Dextrocardia also does not comment on the relative anatomy of the remaining viscera [4]. Rather, the term situs is used to describe the overall visceral arrangement within the thoracic and abdominal cavities [4]. Situs solitus, situs inversus, and situs ambiguous are the three types of situs commonly described within the literature and patient case reports. Situs solitus is typical human anatomy with a right-sided vena cava system connecting to a right-sided systemic right atrium with an right-sided liver. Situs inversus is the exact opposite with a left vena cava system connecting to a left-sided systemic right atrium and the liver on the left. Situs ambiguous differs dramatically and includes an abnormal IVC connection, stomach with indeterminate situs, and a midline liver (Figure 2) [5]. Dextrocardia can and does occur in any one of these three situs settings. Our patient had dextrocardia in the setting of situs inversus. The combination of dextrocardia and situs inversus is referred to as situs inversus totalis and affects approximately 1 in every 10,000 people [4, 6, 7].

One of the greatest implications of both dextrocardia and situs inversus alike is the potential additional anomalies that may occur in alongside either condition. Situs inversus can be associated with cardiac and noncardiac anomalies and include conditions such as duodenal atresia, gastrointestinal malrotation, asplenia, ectopic or horseshoe kidney, various pulmonary abnormalities, ventricular septal defects, atrial septal defects, transposition of the great vessels, double-outlet right ventricle, and more [2, 4–6, 8]. Dextrocardia can be associated with similar conditions including asplenia, polysplenia, and multiple noncyanotic congenital heart defects [9]. However, some sources say situs inversus totalis rarely occurs with other cardiac malformations, while another cites an increase to 3–5% prevalence of congenital heart disease from 1% in the general population [2, 7].

One very unique association occurs between situs inversus totalis and Kartagener's Syndrome. Kartagner's syndrome is a disorder characterized by situs inversus totalis coupled with bronchiectasis and sinusitis [2, 4–6]. This would be of interest and importance to anesthesiologists as mucociliary dysfunction is often a component of Kartagener's syndrome requiring special attention to optimization of pulmonary status prior to anesthetizing the patient. This could be in the form of incentive spirometry, bronchodilators, chest physiotherapy, or potentially antibiotic administration [2, 6]. Care to avoid medications that depress ventilation or ciliary activity should be taken into consideration in patients with situs inversus totalis associated with Kartagener's syndrome [2].

As advancements have been and continued to be made in medical imaging and surgical techniques, the medical community will likely continue to see increases in patients surviving into adulthood with congenital heart diseases in isolation and associated with dextrocardia and/or situs inversus [1, 3]. As an anesthesiologist, the anatomic variation that occurs with these conditions changes the procedural process for many routine preoperative and intraoperative interventions and monitoring techniques. For example, standard monitoring techniques will need to be modified. Transesophageal echocardiography views and maneuvers will differ as we have described above and illustrate in Figure 1. Vessel cannulation location may also vary based upon the type of vascular access required and the unique vasculature arrangement in the patient. Our patient required a left internal jugular central venous catheter to provide direct access to the right atrium. This access site not only ensures direct right atrial access but also avoids injury to the thoracic duct [2, 9]. Additionally, the vectors of myocyte depolarization and repolarization differ with the anatomic variation and require the electrocardiography leads be placed in reverse to avoid a false picture of ischemia [6]. In addition to procedural changes, the associated cardiac and noncardiac malformations and potential past surgeries may add an additional level of complexity to typical physiology. Our case report thus captured a relatively rare anatomic anomaly and demonstrated surgical and anesthetic management alterations implemented to ensure a successful outcome. Successful perioperative management requires anesthesiologists, surgeons, proceduralists, and perioperative care teams to identify, understand, and prepare for alterations and anatomy and pathophysiology that these conditions embody and the potential implications they may have on intraoperative and perioperative management [9].

In summary, dextrocardia with situs inversus and other similar cardiac anatomic abnormalities greatly affects the procedures performed by and management provided by surgeons and anesthesiologists alike. These anomalies also have the potential to be accompanied by further physiologic and anatomic complications that must be carefully assessed and taken into consideration when planning for operative care. Our patient had known dextrocardia with situs inversus and underwent a successful CABG in large part due to the extensive preoperative preparation and planning surrounding the necessary alterations to typical procedural processes and monitoring techniques. Although our case report is limited, in that it only includes one patient, it still demonstrates that it is the responsibility of every member of the healthcare team to ensure patient safety is top priority, and our case demonstrates just how vital careful preparation was in ensuring a successful surgical outcome.
